# Association between acetaminophen use and 30-day mortality in critically ill patients with pulmonary embolism: a retrospective cohort study using the MIMIC-IV database

**DOI:** 10.3389/fphar.2025.1663773

**Published:** 2025-09-02

**Authors:** Xincai Wang, Xiankun Lin, Liman Qiu, Xunwei Tu, Linqian Jiang, Xiuling Shang, Long Huang

**Affiliations:** ^1^ Department of Critical Care Medicine, Fuzhou University Affiliated Provincial Hospital, Fujian Provincial Hospital, Shengli Clinical Medical College of Fujian Medical University, Fuzhou, China; ^2^ The United Innovation of Mengchao Hepatobiliary Technology Key Laboratory of Fujian Province, Mengchao Hepatobiliary Hospital of Fujian Medical University, Fuzhou, China; ^3^ Department of Respiratory and Critical Care Medicine, Fuzhou University Affiliated Provincial Hospital, Fujian Provincial Hospital, Shengli Clinical Medical College of Fujian Medical University, Fuzhou, China

**Keywords:** acetaminophen, pulmonary embolism, intensive care unit, 30-day mortality, propensity score matching, causal mediation analysis

## Abstract

**Purpose:**

Pulmonary embolism (PE) has high mortality rates among critically ill patients. While acetaminophen shows potential therapeutic effects in critical illness, its impact on ICU patients with PE remains unclear. This study evaluated the association between acetaminophen use and 30-day mortality in ICU patients with PE.

**Patients and methods:**

A retrospective cohort study was conducted using the Medical Information Mart for Intensive Care IV (MIMIC-IV) database. Adult patients admitted to the ICU with confirmed PE between 2008 and 2022 were included. The primary exposure was acetaminophen use during the ICU stay, and the primary outcome was 30-day all-cause mortality. To control for confounding factors, propensity score matching (PSM) was applied, along with inverse probability weighting, propensity score adjustment, and E-value analysis to assess result robustness. Causal mediation analysis was performed to evaluate the mediating role of body temperature.

**Results:**

Among 1,983 eligible patients, 1,355 received acetaminophen and 628 did not. After propensity score matching (599 pairs), acetaminophen use was associated with a 31% reduction in 30-day mortality risk (HR 0.69, 95% CI: 0.56–0.85, P = 0.001). This protective effect was more pronounced in patients requiring mechanical ventilation (HR 0.53, 95% CI: 0.34–0.82) and vasopressor support (HR 0.52, 95% CI: 0.32–0.83). Enhanced benefits were also observed in younger patients (<65 years, HR 0.52, 95% CI: 0.31–0.85). Multiple sensitivity analyses yielded consistent results, with E-values ranging from 2.03 to 2.26, suggesting robust resistance to unmeasured confounding. Causal mediation analysis revealed that 54.2% (95% CI: 25.6%–187.5%, P = 0.014) of acetaminophen’s apparent protective effect was mediated through body temperature regulation during hospitalization.

**Conclusion:**

acetaminophen use was associated with reduced 30-day mortality in critically ill patients with PE, with temperature control appearing to play a potential mediating role. These preliminary findings provide hypothesis-generating evidence that warrants validation in prospective randomized controlled trials before clinical implementation.

## Introduction

Pulmonary embolism (PE) represents a major public health concern as the third most lethal cardiovascular disease after myocardial infarction and stroke. A 2021 World Health Organization survey spanning 123 countries reported PE-related mortality rates ranging from 0 to 24 deaths per 100,000 population annually (Barco et al., 2021). The annual incidence of PE is estimated at 0.4%–1.2%, affecting approximately 370,000 individuals in the United States alone and resulting in 60,000 to 100,000 deaths (Konstantinides et al., 2019; Freund et al., 2022). The risk increases substantially with age, with octogenarians experiencing an eight-fold higher incidence compared to individuals aged 40–50 years (Kulka et al., 2021).

Patient outcomes in PE are closely tied to disease severity and treatment conditions. While general inpatient PE mortality is approximately 5.6% (Lüthi-Corridori et al., 2023), this rate could exceed 20% when patients develop hemodynamic instability or shock (Becattini et al., 2024). The prognosis is particularly concerning for ICU patients, where PE-related mortality reaches 18.5%, with a median time to all-cause death of just 6.2 days (Ryll et al., 2024). These statistics underscore the critical importance of early recognition and intervention in severe PE cases. Despite standard anticoagulation therapies, mortality rates remain high among critically ill PE patients, highlighting the need to explore additional therapeutic approaches.

Acetaminophen, also known as paracetamol, is a widely used antipyretic and analgesic that selectively inhibits cyclooxygenase (COX)-1 and COX-2 in the central nervous system (Jaeschke and Ramachandran, 2024; de Sévaux et al., 2023). Its mechanism of action involves reducing COX activity and prostaglandin synthesis (Bruno et al., 2014). Recent research has revealed broader pharmacological effects of acetaminophen. As a potent hemoglobin reductant, it inhibits hemoglobin-mediated oxidation of lipids and other substrates (Domizi et al., 2024), demonstrating potential therapeutic value in critical illness. For instance, a phase 2a clinical trial involving 40 sepsis patients showed that acetaminophen significantly reduced lipid peroxidation biomarkers and improved organ function (Janz et al., 2015). A multicenter retrospective observational study further confirmed reduced mortality among critically ill patients receiving acetaminophen (Suzuki et al., 2015). Of particular interest is acetaminophen’s potential role in thrombotic diseases. Although acetaminophen is not traditionally considered an antiplatelet agent, Munsterhjelm et al. reported it may exert modest, dose-dependent inhibition of platelet aggregation under certain experimental conditions, though findings remain inconsistent and clinically limited (Munsterhjelm et al., 2005). However, findings across the literature remain inconsistent (Driver et al., 2020). It suggests the need for further research into the value of acetaminophen in thromboembolic diseases.

The value of acetaminophen in severe PE patients, particularly those requiring intensive care, remains inadequately evaluated. These patients often present with hemodynamic instability and organ dysfunction, where acetaminophen’s potential antioxidant properties may contribute to outcomes, though evidence in this specific patient population remains limited and requires further investigation (Janz et al., 2015; Suzuki et al., 2015; Ware et al., 2024). However, existing studies have primarily focused on general critical care populations, leaving a gap in knowledge regarding ICU patients with PE.

To address this gap, we conducted a retrospective observational study using the MIMIC-IV database to investigate the association between acetaminophen use and 30-day mortality in ICU patients with PE. We employed propensity score matching to control for confounding factors and conducted multiple sensitivity analyses to verify result robustness. Through systematic evaluation of acetaminophen’s treatment effects across different disease severity levels, we aim to provide important evidence-based guidance for therapeutic strategy selection in ICU patients with PE.

## Materials and methods

### Data source

This study utilized the Medical Information Mart for Intensive Care IV (MIMIC-IV, version 3.1) database, a large, publicly available critical care database containing comprehensive clinical data for over 60,000 ICU admissions at Beth Israel Deaconess Medical Center between 2008 and 2022. The database includes detailed patient information, including demographics, physiological measurements, laboratory test results, diagnostic codes, and medical interventions. The MIMIC-IV database has received ethical approval from the Institutional Review Boards of both MIT and Beth Israel Deaconess Medical Center. All protected health information has been de-identified, eliminating the need for individual informed consent. Author L.H. obtained authorization to access the database (certification number: 11706768). This study follows the STROBE (Strengthening the Reporting of Observational Studies in Epidemiology) guidelines.

### Study population

**FIGURE 1:**
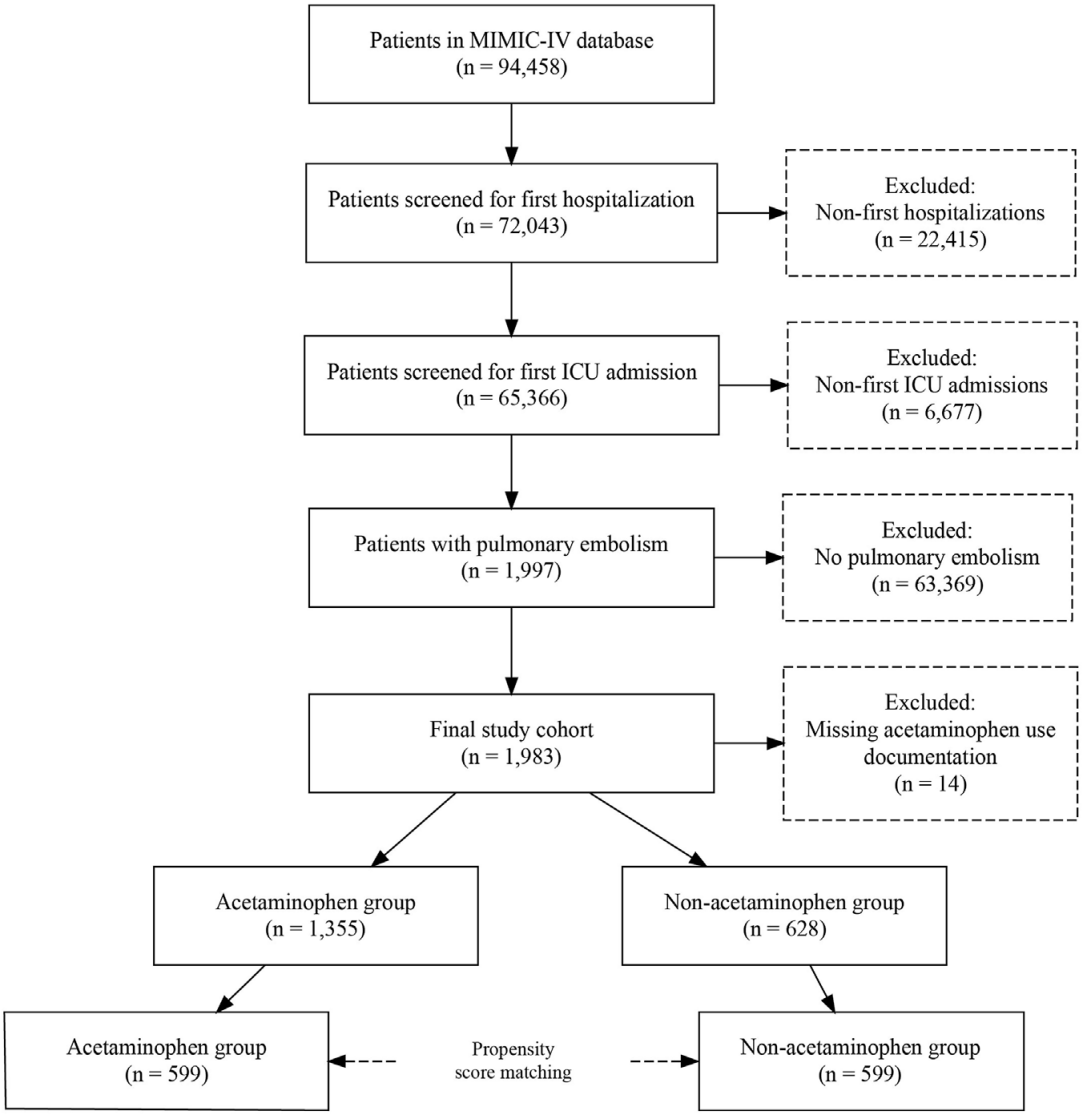
Study population.

Among 94,458 patients in the MIMIC-IV database, we excluded 22,415 non-first hospitalizations and 6,677 non-first ICU admissions, leaving 65,366 patients for screening. Of these, 1,997 patients were diagnosed with PE based on International Classification of Diseases, Ninth Revision (ICD-9) codes (Zhan et al., 2007; Sadeghi et al., 2015). After excluding 14 patients with missing acetaminophen use records, 1,983 patients were included in the final analysis, with 1,355 in the acetaminophen group and 628 in the control group. Following propensity score matching, 599 pairs of patients were analyzed (Figure 1).

### Exposure and covariates

The primary exposure was defined as any acetaminophen use during ICU stay. We selected covariates based on previous research and clinical experience, encompassing a comprehensive set of clinical parameters. These included demographic characteristics (age and sex), physiological measurements during ICU stay (mean heart rate, mean arterial pressure, respiratory rate, and oxygen saturation), and laboratory values obtained within the first 24 h of ICU admission (hemoglobin, platelet count, white blood cell count, creatinine, blood urea nitrogen, sodium, and potassium). Relevant comorbidities were also considered, including heart failure, peripheral vascular disease, cerebrovascular disease, chronic pulmonary disease, diabetes, kidney disease, and malignancy. To account for illness severity, we incorporated disease severity scores (Sequential Organ Failure Assessment [SOFA] and Acute Physiology Score III [APSIII]) calculated at ICU admission, along with key therapeutic interventions such as vasopressor use and mechanical ventilation.

### Primary outcome

The primary outcome was 30-day all-cause mortality following ICU admission. Patient survival status was tracked through hospital records and the Social Security Administration Death Master File.

### Statistical analysis

We performed descriptive analyses for all study subjects. Categorical variables were presented as counts and percentages, and continuous variables as means and standard deviations. Between-group comparisons used chi-square tests for categorical variables and t-tests for continuous variables. To reduce treatment allocation bias and confounding effects, we employed propensity score matching. Propensity scores were calculated using a Cox proportional hazards regression model with survival time as the outcome variable, incorporating all aforementioned covariates. We used 1:1 nearest neighbor matching with a caliper width of 0.01. Matching balance was assessed using standardized mean differences (SMD), with SMD<0.1 considered acceptable. In the matched cohort, Kaplan-Meier curves were constructed for 30-day survival, with between-group differences assessed using log-rank tests. Sensitivity analyses included inverse probability weighting and propensity score adjustment. Cox proportional hazards regression models with robust variance estimation were used to calculate 30-day mortality hazard ratios (HR) and 95% confidence intervals. The potential impact of unmeasured confounding was evaluated using E-value analysis. To assess mediation effects, we utilized a comprehensive analytical framework incorporating the Sobel test, Bootstrap resampling technique, and quasi-Bayesian Monte Carlo estimation with 1,000 replications based on normal distribution assumptions. All analyses were performed using R software version 4.0.3 (R Foundation for Statistical Computing, Vienna, Austria). Two-sided P-values <0.05 were considered statistically significant. Missing values, which occurred in <5% of variables, were handled using single imputation based on other available covariate information. Mean body temperature during ICU stay was calculated for each patient from all available temperature measurements. The association between acetaminophen use and body temperature was evaluated using Spearman’s rank correlation coefficient due to non-normal distribution of temperature data. Between-group comparisons were performed using the Mann-Whitney U test. Statistical significance was set at P < 0.05. Causal mediation analysis (CMA) is a method to differentiate the total effect of a treatment into direct and indirect effects (Rijnhart et al., 2021). The indirect effect on the outcome is mediated via a mediator. The analysis produces an average causal mediation effect (ACME), average direct effect (ADE) and total effect. Given that acetaminophen is commonly used for fever reduction in clinical practice, we explored whether the effect of acetaminophen use on 30-day mortality is proportionally mediated by mean body temperature during hospitalization. We used CMA to characterize the causality relationship between acetaminophen administration, mean body temperature during hospitalization, and mortality outcomes in our retrospective study.

## Results

### Baseline characteristics

Before propensity score matching, significant differences existed between the acetaminophen (n = 1,355) and control groups (n = 628) in multiple baseline characteristics (Table 1). The acetaminophen group was younger (62.61 ± 17.49 vs. 66.34 ± 15.37 years) with higher disease severity, as evidenced by higher SOFA scores (median [IQR]: 3.0 [1.0, 6.0] vs. 3.0 [1.0, 5.0]) and APSIII scores (52.13 ± 26.40 vs. 48.07 ± 25.45). These patients required more organ support, including mechanical ventilation (44.0% vs. 24.7%) and vasopressors (31.3% vs. 19.4%). Regarding comorbidities, the acetaminophen group had a higher prevalence of cerebrovascular disease (15.9% vs. 10.0%) but lower malignancy rates (23.7% vs. 33.1%).

**TABLE 1:** Baseline characteristics before and after propensity score matching.

	Unmatched patients		SMD	Matched patients		SMD
	Control (n = 628)	Acetaminophen (n = 1,355)		Control (n = 599)	Acetaminophen (n = 599)	
Demographics						
Age, years	66.34 (15.37)	62.61 (17.49)	0.226	66.31 (15.39)	66.06 (16.33)	0.016
Male sex	308 (50.7)	645 (48.4)	0.048	301 (50.3)	298 (49.7)	0.010
Physiological Parameters						
Heart rate, bpm	92.34 (17.01)	91.49 (17.61)	0.049	92.32 (17.00)	93.04 (17.73)	0.042
Mean BP, mmHg	80.06 (11.66)	81.23 (11.10)	0.103	80.15 (11.67)	80.70 (11.01)	0.049
Respiratory rate	20.79 (4.32)	21.16 (4.42)	0.082	20.79 (4.30)	20.91 (4.19)	0.028
SpO2, %	95.66 (4.38)	96.29 (2.48)	0.177	95.89 (3.15)	95.89 (2.83)	<0.001
Laboratory Values						
Hemoglobin, g/dL	10.19 (2.45)	10.33 (2.36)	0.058	10.21 (2.44)	10.20 (2.32)	0.004
Platelets, ×10^3^/μL	218.95 (149.34)	209.01 (112.53)	0.075	216.66 (135.27)	218.70 (123.62)	0.016
WBC, ×10^3^/μL	13.67 (13.29)	15.64 (15.03)	0.138	13.67 (13.35)	13.57 (7.18)	0.009
Creatinine, mg/dL	1.42 (1.92)	1.31 (1.26)	0.068	1.36 (1.29)	1.38 (1.37)	0.012
BUN, mg/dL	26.28 (20.88)	25.21 (20.18)	0.052	26.25 (20.88)	25.83 (21.16)	0.020
Sodium, mEq/L	136.38 (5.16)	136.70 (5.19)	0.061	136.41 (5.17)	136.45 (5.09)	0.008
Potassium, mEq/L	4.53 (0.82)	4.57 (0.85)	0.046	4.53 (0.82)	4.53 (0.81)	0.003
Comorbidities						
Heart failure	134 (22.1)	328 (24.6)	0.059	134 (22.4)	134 (22.4)	<0.001
Peripheral vascular disease	39 (6.4)	97 (7.3)	0.034	38 (6.3)	42 (7.0)	0.027
Cerebrovascular disease	61 (10.0)	212 (15.9)	0.175	61 (10.2)	57 (9.5)	0.022
Chronic pulmonary disease	178 (29.3)	387 (29.0)	0.007	177 (29.5)	186 (31.1)	0.033
Diabetes	155 (25.5)	303 (22.7)	0.066	151 (25.2)	157 (26.2)	0.023
Renal disease	77 (12.7)	153 (11.5)	0.037	76 (12.7)	76 (12.7)	<0.001
Malignant cancer	201 (33.1)	316 (23.7)	0.210	197 (32.9)	185 (30.9)	0.043
Severity Scores						
SOFA	3.0 (1.0, 5.0)	3.0 (1.0, 6.0)	0.126	3.0 (1.0, 5.0)	3.0 (1.0, 5.0)	0.029
APSIII	48.07 (25.45)	52.13 (26.40)	0.157	48.00 (25.51)	47.14 (23.93)	0.035
Treatment Parameters						
Vasopressor use	118 (19.4)	417 (31.3)	0.274	115 (19.2)	110 (18.4)	0.021
Mechanical ventilation	150 (24.7)	587 (44.0)	0.415	149 (24.9)	150 (25.0)	0.004

Data are presented as mean (SD) or n (%).

SMD, standardized mean difference; values > 0.1 indicate potentially meaningful differences between groups.

bpm, beats per minute; BP, blood pressure; WBC, white blood cell count; BUN, blood urea nitrogen; SOFA, sequential organ failure assessment; APSIII, Acute Physiology Score III.

After 1:1 propensity score matching, 599 pairs of patients were included. All baseline characteristics achieved good balance with SMD<0.1. Specifically, the groups showed minimal differences in key prognostic factors such as age (66.06 ± 16.33 vs. 66.31 ± 15.39 years), SOFA scores (median [IQR]: 3.0 [1.0, 5.0] vs. 3.0 [1.0, 5.0]), and mechanical ventilation use (25.0% vs. 24.9%).

### Primary outcome analysis

**FIGURE 2:**
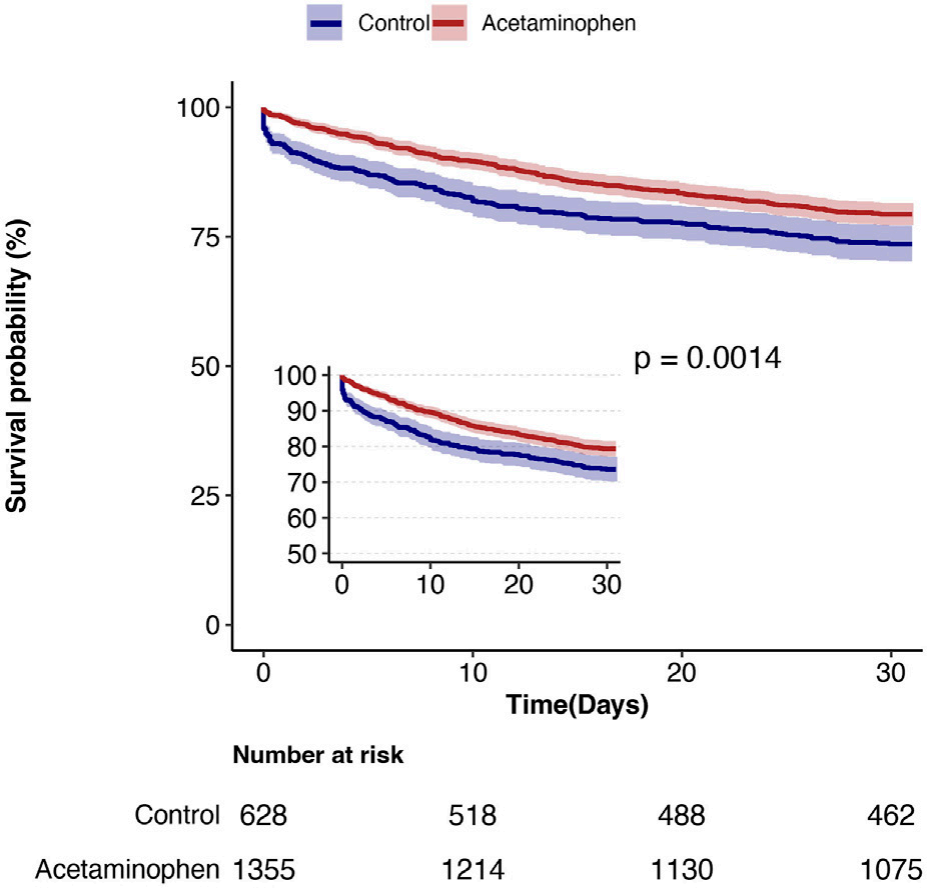
Kaplan-Meier survival curves.

During the 30-day follow-up, mortality was significantly lower in the acetaminophen group compared to the control group (20.7% vs. 26.4%). Cox proportional hazards regression analysis showed that acetaminophen use was associated with a 27% reduction in 30-day mortality risk in unadjusted analysis (HR = 0.73, 95% CI: 0.60–0.89, P = 0.001). After adjusting for all covariates in Table 1, this protective association strengthened (HR = 0.69, 95% CI: 0.56–0.85, P = 0.001), indicating a 31% reduction in mortality risk. The E-value of 2.26 (95% CI: 1.50) suggests robust resistance to unmeasured confounding (Table 2). Kaplan-Meier survival curves (Figure 2) demonstrated significantly better survival in the acetaminophen group throughout the follow-up period (log-rank test, P = 0.001).

**TABLE 2:** Associations between acetaminophen use and 30-day mortality in different analyses.

Analysis	30-day Mortality (%)	P-value	E-value	E-value (CI)
No. of events/no. of patients at risk (%)				
No acetaminophen use	166/628 (26.4)			
Acetaminophen use	280/1,355 (20.7)			
Crude analysis - hazard ratio (95% CI)	0.73 (0.60–0.89)	0.001	2.03	1.38
Multivariable analysis - hazard ratio (95% CI)^a^	0.69 (0.56–0.85)	0.001	2.26	1.50
With inverse probability weighting^b^	0.69 (0.57–0.83)	0.001	2.26	1.63
With matching^c^	0.69 (0.54–0.88)	0.003	2.26	1.45
Adjusted for propensity score^d^	0.73 (0.60–0.90)	0.003	2.03	1.39

^a^ Shown is the hazard ratio from the multivariable Cox proportional hazards model, adjusted for all covariates in Table 1.

^b^ Shown is the primary analysis with a hazard ratio from the multivariable Cox proportional-hazards model with inverse probability weighting according to the propensity score.

^c^ Shown is the hazard ratio from a multivariable Cox proportional-hazards model with matching according to the propensity score. The analysis included 599 matched pairs.

^d^ Shown is the hazard ratio from a multivariable Cox proportional-hazards model with additional adjustment for the propensity score.

### Subgroup analysis

**FIGURE 3:**
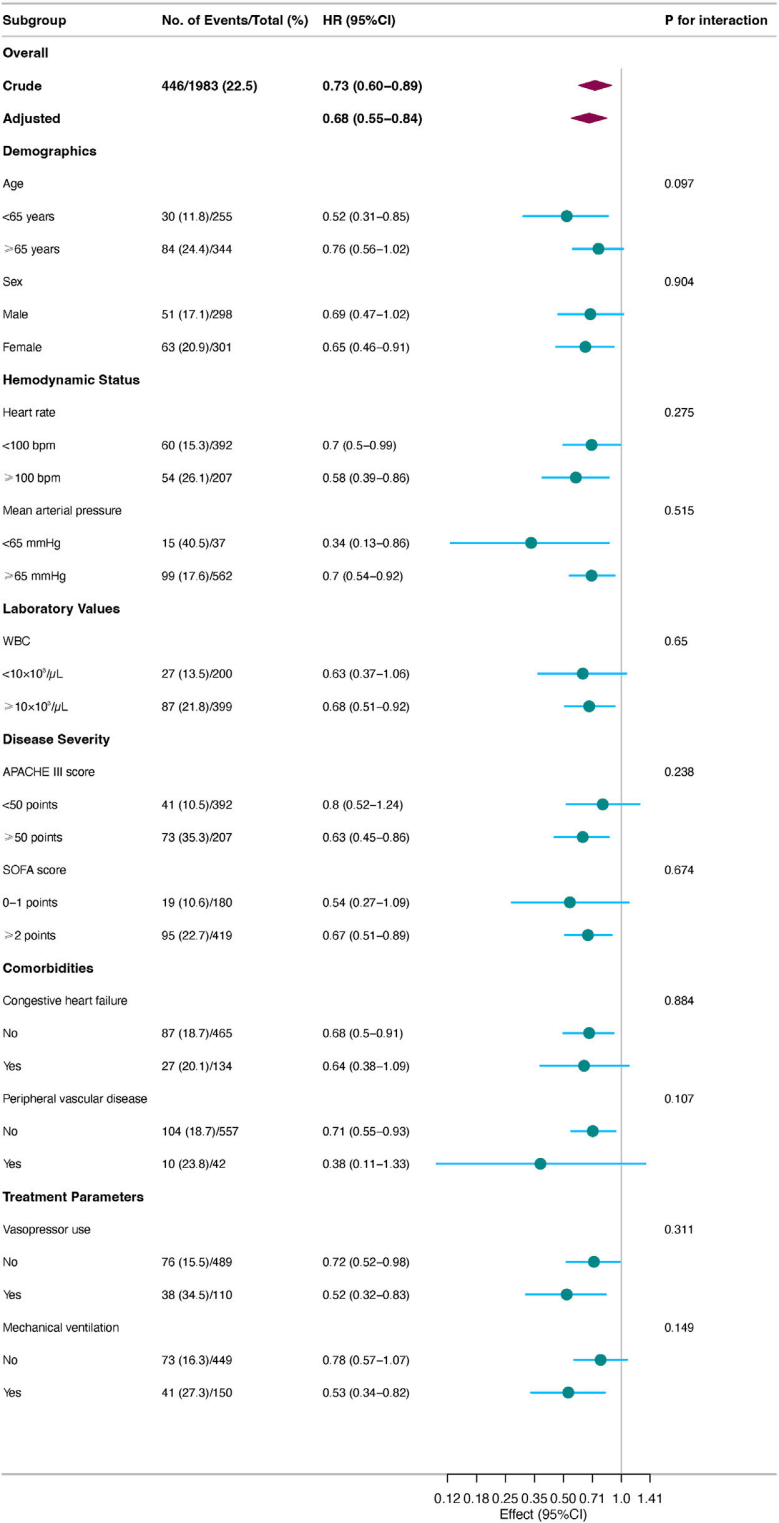
Prespecified subgroup analyses.

Prespecified subgroup analyses (Figure 3) revealed that acetaminophen’s protective effect was more pronounced in patients with higher disease severity. Specifically, significant mortality risk reductions were observed in patients with APACHE III scores ≥50 (HR = 0.63, 95% CI: 0.45–0.86), those requiring mechanical ventilation (HR = 0.53, 95% CI: 0.34–0.82), and those requiring vasopressor support (HR = 0.52, 95% CI: 0.32–0.83). Enhanced benefits were also observed in patients with heart rates ≥100 beats/min (HR = 0.56, 95% CI: 0.39–0.86) and younger patients (<65 years, HR = 0.52, 95% CI: 0.31–0.85). Notably, interaction tests for all prespecified subgroups were non-significant (all P-interaction>0.05), suggesting consistent treatment effects across subgroups.

### Sensitivity analysis

Multiple sensitivity analyses (Table 2) demonstrated robust results. Consistent findings were obtained using inverse probability weighting (HR = 0.69, 95% CI: 0.57–0.83), propensity score matching (HR = 0.69, 95% CI: 0.54–0.88), and propensity score adjustment (HR = 0.73, 95% CI: 0.60–0.90), all indicating significantly reduced mortality risk with acetaminophen use (all P-values ≤0.003). E-values ranged from 2.03 to 2.26, indicating strong robustness against unmeasured confounding across all analytical approaches. E-value analysis (Table 3) showed that an unmeasured confounder with relative risks of at least 2.26 for both exposure and outcome would be needed to fully explain the observed association (RR = 1.45, 95% CI: 1.12–1.67). An unmeasured confounder with a strength of 1.50 would be required to invalidate the lower confidence interval limit, suggesting strong robustness to unmeasured confounding.

**TABLE 3:** Risk estimates and E-value analysis.

Measure	Point estimate	95% CI
Risk Ratio	1.45	1.12to 1.67
E-value	2.26	1.50 to NA^a^

^a^ NA, not applicable for upper confidence limit.

### Association between acetaminophen use and body temperature

**FIGURE 4:**
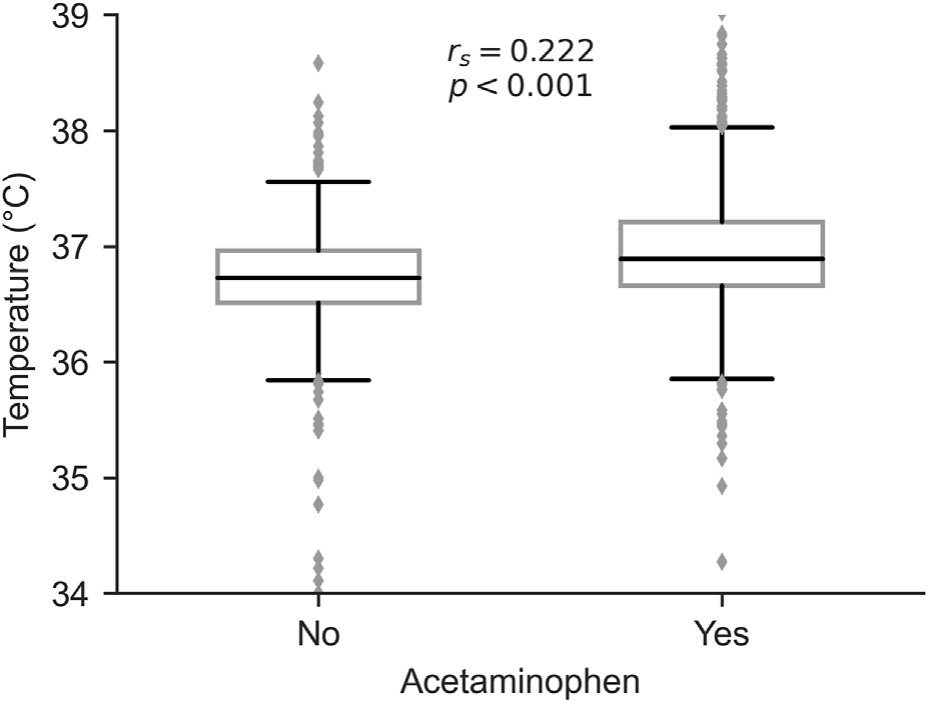
Association between acetaminophen use and mean body temperature during ICU stay.

To investigate the clinical indications for acetaminophen administration, we analyzed the relationship between acetaminophen use and mean body temperature during ICU stay. Patients who received acetaminophen had significantly higher mean body temperatures compared to those who did not (median [IQR]: 37.0 [36.8–37.2] °C vs. 36.8 [36.5–37.0] °C, P < 0.001, Mann-Whitney U test). Spearman’s rank correlation analysis revealed a significant positive correlation between acetaminophen use and mean body temperature (rs = 0.222, P < 0.001, Figure 4), suggesting that fever management was a primary indication for acetaminophen administration in our cohort.

### Causal mediation analysis

**FIGURE 5:**
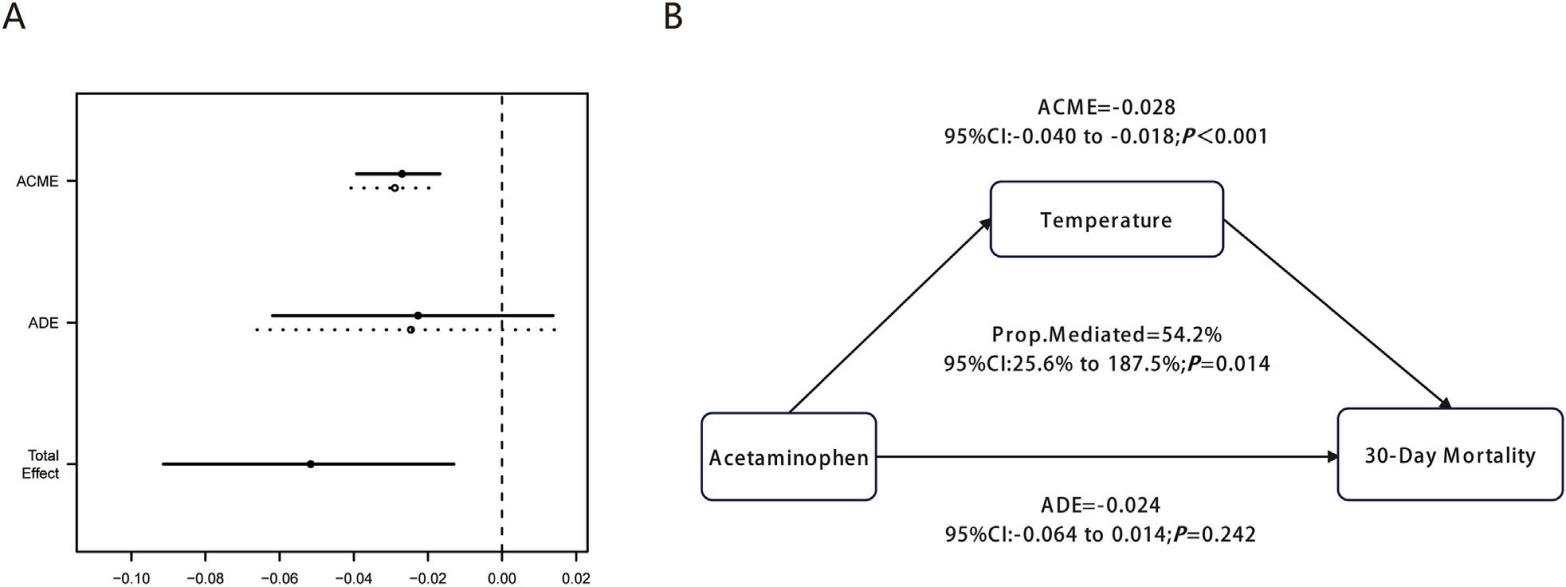
Causal mediation analysis for mean body temperature during hospitalization. **(A)** shows the graphical representation of ACME, ADE, and Total Effect with confidence intervals. **(B)** illustrates the mediation diagram showing the causal relationship between Acetaminophen, Temperature, and 30-Day Mortality with corresponding statistical values.

To explore the potential mechanisms underlying acetaminophen’s protective effect on 30-day mortality, we conducted causal mediation analysis using body temperature as the mediating variable. The analysis revealed that mean body temperature during hospitalization significantly mediated the relationship between acetaminophen use and improved survival outcomes (Figure 5).

The mediation analysis demonstrated a significant average causal mediation effect (ACME) of −0.028 (95% CI: 0.040 to −0.018, P < 0.001), indicating that acetaminophen’s protective effect was substantially mediated through body temperature during hospitalization. This finding confirms the biological plausibility of the temperature-mediated pathway in reducing 30-day mortality among ICU patients with pulmonary embolism.

Notably, 54.2% (95% CI: 25.6%–187.5%, P = 0.014) of acetaminophen’s total protective effect on 30-day mortality was mediated through body temperature during hospitalization. In contrast, the average direct effect (ADE) was −0.024 (95% CI: 0.064 to 0.014, P = 0.242), which was not statistically significant. This finding suggests that acetaminophen’s protective effect in PE patients operates primarily through t body temperature during hospitalization rather than direct mechanisms independent of temperature changes, highlighting the critical role of fever control in the management of critically ill patients with pulmonary embolism.

## Discussion

In this retrospective cohort study utilizing the MIMIC-IV critical care database, we systematically evaluated the association between acetaminophen use and 30-day mortality in ICU patients with PE. Through analysis of 1,983 consecutive admissions and comprehensive propensity score matching with multiple sensitivity analyses, we found that acetaminophen use was associated with a significant reduction in 30-day mortality risk (HR 0.69, 95% CI 0.56–0.85), corresponding to an approximately 31.6% decrease in 30-day mortality compared to non-use. This protective association demonstrated good consistency across multiple prespecified subgroups and was further validated through E-value analysis. Notably, our observed 30-day mortality rates were higher than those reported in two previous studies: a multicenter retrospective observational study of 273 ICU PE patients (2014–2018) reporting 15.3% mortality (İdin et al., 2021), and a prospective cohort study of 1,015 high-risk PE patients (2015–2019) reporting 10.0% (Mirambeaux et al., 2019). This disparity likely reflects our study population’s characteristics, specifically first-time ICU admissions with severe PE.

Acetaminophen exhibits multiple pharmacological effects, including antipyretic, analgesic, and antioxidant properties, with a well-established safety profile at therapeutic doses. Despite its half-century clinical use, many of its disease-specific mechanisms remain unclear (Jóźwiak-Bebenista and Nowak, 2014). Our finding of reduced 30-day mortality risk with acetaminophen use was robust across multiple sensitivity analyses and E-value assessment. This aligns with another MIMIC database retrospective study showing reduced 30-day mortality in critically ill patients receiving acetaminophen (Sun et al., 2024). However, a large cohort study of 700 patients found no significant effect on 28-day survival (Young et al., 2015), possibly due to their focus on febrile patients with known or suspected infection rather than ICU patients with PE. Thus, acetaminophen’s efficacy may depend on underlying pathophysiological mechanisms, with varying effects across different critical illness populations. Our subgroup analyses revealed stronger protective effects in high-risk patients, particularly those with APACHE III scores ≥50, respiratory failure requiring mechanical ventilation, and hemodynamic instability requiring vasopressor support. This aligns with the ASTER randomized clinical trial, where acetaminophen patients showed significant improvements in respiratory and coagulation SOFA scores (Ware et al., 2024). This finding provides preliminary evidence that acetaminophen may potentially improve PE patient outcomes through multiple organ system effects, though further validation is required.

To provide clinical context for our findings and examine the underlying mechanisms of acetaminophen’s protective effects, we conducted a supplementary analysis investigating the relationship between acetaminophen administration and body temperature in our cohort. This analysis revealed a positive correlation between acetaminophen use and mean body temperature during ICU stay (rs = 0.222, P < 0.001), suggesting that fever management was likely a primary indication for acetaminophen administration in our patients. This finding provides biological plausibility for our temperature-mediation hypothesis and helps explain why temperature control emerged as a significant mediator in our causal analysis. Notably, our subgroup analyses demonstrated greater acetaminophen benefit in patients with elevated white blood cell counts (≥10 × 10^3^/μL, HR = 0.68, P = 0.012) and higher SOFA scores (≥2 points, HR = 0.67, P = 0.005), populations that likely had higher prevalence of infection-related fever and systemic inflammation. However, we acknowledge that temperature regulation represents only one potential pathway through which acetaminophen may exert its protective effects. While our mediation analysis suggested that a substantial proportion (54.2%) of the benefit was mediated through temperature control, the complexity of critical illness suggests that multiple mechanisms likely contribute to the observed outcomes, including acetaminophen’s antioxidant properties, anti-inflammatory effects, and potential interactions with hemodynamic and metabolic pathways independent of fever reduction.

The observed associations might potentially involve the following mechanisms, although causal relationships cannot be established from our observational data. First, PE patients are in a pathological state of oxidative stress, with platelet mitochondrial oxygen consumption and ROS production increased by >30% in acute intermediate-risk PE patients (Rezania et al., 2017). Excessive ROS causes DNA damage and lipid peroxidation, and induces fever through excessive catabolism. The antipyretic effect of acetaminophen is crucial in this pathological state, as hyperthermia significantly increases metabolic burden and oxygen consumption, impairs vital organ function, and increases mortality risk (Drewry and Mohr, 2022). Acetaminophen effectively controls fever and alleviates metabolic stress through central COX inhibition, which reduces prostaglandin synthesis (de Sévaux et al., 2023; Bruno et al., 2014). Through causal mediation analysis (CMA), we found that body temperature regulation during hospital stay may represent a potentially important mediating pathway for acetaminophen’s apparent protective associations with 30-day mortality in PE patients, though other direct mechanisms cannot be definitively excluded. Muhammad Saad et al.'s study found in univariate analysis that febrile PE patients had higher in-hospital mortality compared with afebrile PE patients (mortality rate: 22.0% vs. 10.4%) (Saad et al., 2018). Second, acetaminophen can protect vascular endothelial function through its anti-oxidant and anti-inflammatory properties (Tripathy and Grammas, 2009). Our observation of greater benefit in patients requiring mechanical ventilation aligns with previous findings suggesting acetaminophen may improve respiratory function by preventing free hemoglobin-induced alveolar-capillary barrier dysfunction (Ware et al., 2024; Shaver et al., 2018), explaining the enhanced benefit in patients with respiratory failure. Third, PE pathogenesis may involve platelet activation and aggregation-induced thrombosis. A large case-control study (n = 809) found significant associations between acetaminophen use and thrombocytopenia (Gonzalez-Valcarcel et al., 2016). This may occur through dose-dependent COX-1 inhibition reducing thromboxane A_2_ synthesis and platelet aggregation (Kao et al., 2022; Kovačič et al., 2019). However, it is important to note that acetaminophen’s antiplatelet effects remain inconsistent across studies and are generally considered modest compared to dedicated antiplatelet agents. These potential effects may contribute to reduced pulmonary vascular thrombosis in PE patients, though further research is needed to establish definitive antiplatelet properties of acetaminophen. Additionally, acetaminophen may improve hemodynamics through COX inhibition (MacIntyre et al., 2022; Spence et al., 2022), without increasing stroke or myocardial infarction risk (Yi et al., 2024). The observed mortality reduction in hemodynamically unstable high-risk PE patients may relate to improved organ and tissue perfusion.

Our findings provide preliminary observational evidence that warrants further investigation in ICU PE management. The observed associations suggest that the relationship between temperature control and outcomes in PE patients may merit evaluation in prospective studies, particularly regarding potential mechanisms involving oxidative stress and catabolism. Nevertheless, prospective randomized controlled trials are essential to establish causality before clinical guideline implementation. Future investigations should first establish whether a causal relationship exists between acetaminophen use and improved outcomes through adequately powered randomized controlled trials. If causality is established, subsequent studies could then evaluate optimal timing, dosing regimens, and potential interactions with standard PE therapies (Yang et al., 2024).

This study has several strengths. The MIMIC-IV database provided adequate sample size and detailed clinical information for comprehensive patient characteristic and outcome assessment. Our rigorous propensity score matching methodology, multiple sensitivity analyses (including inverse probability weighting and propensity score adjustment), and E-value analysis enhanced result reliability. Extensive prespecified subgroup analyses helped identify populations most likely to benefit while improving clinical interpretability. The inclusion of multiple disease severity indicators and organ function parameters enabled comprehensive evaluation of acetaminophen’s treatment effects across different clinical contexts.

Several important limitations warrant discussion. As a retrospective observational study using single-center MIMIC-IV data, our findings cannot establish causality between acetaminophen use and improved outcomes, despite robust statistical controls for measured confounding. To address these limitations and provide definitive evidence, future prospective randomized controlled trials should employ 90-day all-cause mortality as the primary endpoint, which better captures both acute management and longer-term recovery outcomes in critically ill PE patients, with pre-specified subgroup stratification based on disease severity markers identified in our analysis where enhanced acetaminophen benefits were observed. Confounding by indication represents a critical concern, as acetaminophen is typically prescribed for specific clinical indications that may themselves be associated with prognosis. While the MIMIC-IV database contains detailed medication administration records, our binary exposure classification (any acetaminophen use vs. none) was adopted due to methodological challenges including high missing data rates in dosing documentation, heterogeneous prescribing practices across the study period, and insufficient statistical power for meaningful dose-stratified analyses. This approach may obscure important dose-response relationships and potential ‘therapeutic window’ effects. Future prospective studies should implement standardized dosing protocols with systematic documentation to enable robust dose-stratified analyses and identify optimal therapeutic windows. An important methodological limitation is our inability to conduct ideal negative control exposure analysis. Despite attempting to use other antipyretic medications as controls, we encountered severe sample size limitations and co-medication confounding issues. This limits our ability to separate acetaminophen-specific effects from general antipyretic actions, further emphasizing the uncertainty around our temperature-mediation mechanism conclusions.

Furthermore, the variability in dosing documentation practices and incomplete capture of cumulative exposure prevent reliable dose-stratified analysis that would be necessary to explore potential “J-shaped curve” relationships, including hepatotoxicity risks at higher doses. PE diagnosis relied solely on ICD-9 codes without integration of imaging or laboratory gold standards, introducing risk of misclassification. However, this diagnostic approach reflects standard methodology in large-scale database studies, and previous validation studies have demonstrated the diagnostic validity of ICD-9 codes for PE identification in similar clinical databases. Any potential misclassification would likely be non-differential, potentially biasing results toward the null hypothesis. The MIMIC-IV database, like most large clinical databases, was not designed with systematic pharmacovigilance as a primary objective, resulting in limited structured adverse event documentation that prevents comprehensive risk-benefit assessment. This represents a common challenge in retrospective database research rather than a specific study limitation. Residual confounding from unmeasured variables (frailty, detailed comorbidity severity, care preferences) likely persists despite sensitivity analyses, and generalizability may be limited given the U.S. academic medical center setting and temporal span (2008–2022). These limitations collectively emphasize that our findings provide valuable preliminary evidence following the established research paradigm where observational studies identify potential therapeutic signals and inform clinical trial design, requiring validation through adequately powered, prospective, randomized controlled trials with systematic safety monitoring before any clinical practice recommendations can be made.

## Conclusion

Acetaminophen use was associated with reduced 30-day mortality in critically ill patients with PE, with temperature control appearing to play a potential mediating role. These preliminary findings provide hypothesis-generating evidence that warrants validation in prospective randomized controlled trials before clinical implementation.

## Data Availability

The raw data supporting the conclusions of this article will be made available by the authors, without undue reservation.
